# DOES THE ASSOCIATION OF TACROLIMUS AND MYCOPHENOLATE MOFETIL CHANGE THE HEALING OF THE ABDOMINAL WALL? STUDY IN RATS SUBMITTED TO ISCHEMIA AND KIDNEY REPERFUSION

**DOI:** 10.1590/0102-672020200004e1551

**Published:** 2021-01-25

**Authors:** André Luís Conde WATANABE, Jorge Eduardo Fouto MATIAS

**Affiliations:** 1 Area of Surgical Clinic, Faculty of Medicine, University of Brasília, Brasília, DF, Brazil; 2 Department of Surgery, Federal University of Paraná, Curitiba, PR, Brazil

**Keywords:** Wound healing, Abdominal wall, Tacrolimus, Mycophenolic acid, Cicatrização, Parede abdominal, Tacrolimo, Ácido Micofenólico

## Abstract

**Background:**

Tacrolimus and mycophenolate mofetil are immunosuppressive agents widely used on the postoperative period of the transplants.

**Aim:**

To evaluate the influence of the association of them on the abdominal wall healing in rats.

**Methods:**

Thirty-six Wistar rats were randomly assigned in three groups of 12. On the early postoperative period, four of the control group and three of the experimental groups died. The three groups were nominated as follow: control group (GC, n=8); group I (GI, n=11, standard operation, mycophenolate mofetil and tacrolimus); group II (GII, n=10, standard operation, mycophenolate mofetil and tacrolimus). The standard operation consisted of right total nephrectomy and 20 min ischemia of the left kidney followed by reperfusion. Both NaCl 0.9% and the immunosuppressive agents were administered starting on the first postoperative day and continuing daily until the day of death on the 14^th^ day. On the day of their deaths, two strips of the anterior abdominal wall were collected and submitted to breaking strength measurement and histological examination.

**Results:**

There were no significant differences in wound infection rates (p=0,175), in the breaking strength measurement and in the histological examination among the three groups.

**Conclusion:**

The combination of the immunosuppressive agents used in the study associated with renal ischemia and reperfusion does not interfere in the abdominal wall healing of rats.

## RESUMO

**Racional:**

O tacrolimus e o micofenolato mofetil são imunossupressores amplamente utilizados no pós-operatório dos transplantes de órgãos.

**Objetivo:**

Avaliar os efeitos deles sobre a cicatrização da parede abdominal em ratos.

**Métodos:**

Foram utilizados 36 ratos Wistar, distribuídos aleatoriamente em três grupos de 12. No pós-operatório imediato, quatro do grupo controle e três do grupo experimentação morreram. Os três grupos receberam as seguintes denominações: grupo controle (GC, n=8); grupo I (GI, n=11, operação-padrão, micofenolato mofetil e tacrolimus); grupo II (GII, n=10, operação-padrão, micofenolato mofetil e tacrolimus). A operação-padrão consistiu de nefrectomia total à direita, isquemia durante 20 min seguida de reperfusão do rim esquerdo. Solução de NaCl 0,9% e micofenolato mofetil + tracolimus foram administradas a partir do 1° dia do pós-operatório e mantidas até o dia do sacrifício dos animais, no 14° dia. Na data do sacrifício, foram retirados dois fragmentos da parede abdominal para análise da resistência à ruptura e exame histológico.

**Resultados:**

Não houve diferença estatisticamente significativa no índice de infecção de ferida operatória (p=0,175), nos valores de resistência de ruptura e nos achados histopatológicos entre os três grupos de animais.

**Conclusão:**

Os esquemas de imunossupressão empregados associados ao fenômeno da isquemia-reperfusão renal não induzem fraqueza significativa da cicatriz da parede abdominal em ratos no 14° dia de pós-operatório.

## INTRODUCTION

The significant increase in the number of organ transplants has required a better understanding of its early and late complications. In the postoperative period of solid organ transplants, high doses of immunosuppressants are administered early to prevent acute rejection. However, these agents inhibit the immune response that plays an important role in the inflammatory and cellular phase of healing and may increase the risk of dehiscence and incisional hernia. In addition, immunosuppressants have been associated with infectious complications at the surgical site, which can further increase the risk of dehiscence^14^. Tacrolimus and mycophenolate mofetil started to play a prominent role in immunosuppression schemes, being widely used. Thus, it became interesting to know the adverse effects of this association, especially in the healing process, since these immunosuppressants are started early in the postoperative period.

Surgical wound healing disorders were observed in 4-50% of kidney transplant recipients, under different immunosuppression regimes^28^. The use of immunosuppressive agents has been associated with complications in the wound healing process; however, experimental and clinical studies have produced contradictory results, demonstrating the need for additional research to assist daily clinical practice^16^. Fikatas et al.^3^ reported that there is still no understanding of the effect of immunosuppressants on the development of incisional hernia, which requires further clarification by complementary studies.

Thus, the aim of this research was to evaluate the effects of the association of immunosuppressive agents tacrolimus and mycophenolate mofetil on the healing of the abdominal wall in rats submitted to total nephrectomy on the right and ischemia and reperfusion of the left kidney.

## METHODS

This research was approved by the Ethics Committee on the Use of Animals of the Biological Sciences Sector of the Federal University of Paraná, Brazil (n° 386b).

Thirty-six male Wistar rats, with body weight between 180-410 g, were used, randomly distributed in a control group and two experimental groups with 12 animals each. In the immediate postoperative period, four animals from the control group and three from the experiment died from anesthetic or operative complications and were excluded from the analysis. The three groups received the following names with the respective numbers of animals: control group (GC), eight rats were submitted to the standard operation and to the administration of 0.9% sodium chloride solution; group I (GI), 11 animals with standard operation, administration of mycophenolate mofetil 20 mg/kg/day and tacrolimus 1 mg/kg/day; group II (GII), 10 animals with standard operation and administration of mycophenolate mofetil 20 mg/kg/day and tacrolimus 0.5 mg/kg/ day. The standard operation consisted of median laparotomy, starting at the xiphoid process until reaching the pubis, to access the abdominal cavity. Initially, total nephrectomy was performed on the right and, afterwards, ischemia for 20 min followed by reperfusion of the left kidney to reproduce a clinical model of kidney transplantation. The synthesis of the abdominal wall was performed in a single musculoaponeurotic plane, without including the peritoneum, using a simple continuous suture type suture with 4.0 mononylon thread. Then, the skin was closed using a simple continuous suture type suture with 4.0 mononylon thread. NaCl 0.9% solution and mycophenolate mofetil+tracolimus were administered by gavage in a single daily dose with a total volume of 0.5 ml. The administration of these solutions was started on the 1^st^ postoperative day and maintained until the day of sacrifice, on the 14^th^ postoperative day. At that time, blood samples were collected from each animal to measure serum levels of albumin and tacrolimus in whole blood, as previously described^27^.

After the macroscopic evaluation of the median scar for diagnosis of surgical wound infection, a 3 cm wide and 4 cm long rectangular fragment of the abdominal wall was removed, containing the surgical scar in its central portion. After careful removal of the skin, two equal fragments of 1 cm in width and 3 cm in length were obtained through incisions parallel and perpendicular to the surgical scar. The cranial fragment was fixed in a 10% buffered formaldehyde solution for histopathological study. In the caudal fragment of the abdominal wall, the study of rupture resistance was performed immediately after resection and division of the flap. To determine this resistance, the EMIC^®^ tensile test equipment, model DL500MF, was used. The intensity of the force required to completely rupture the surgical scar on the abdominal wall was measured in kilogram-force (Kgf). The speed used in the test was 50 mm per minute.

The histopathological study was carried out by means of optical microscopy by a pathologist who was unaware to which group of animals the material analyzed belonged. Microscopic analysis was performed in a 4.0 mm thick histological section perpendicular to the suture line, stained using the H&E technique and Masson’s trichrome method. In the microscopic evaluation, the following tissue changes were analyzed: cell infiltration, vascular neoformation, focal necrosis, vascular congestion, edema, hemorrhage and fibrosis. Regarding the intensity of the changes, the following classification was attributed: absent (-); light (+); moderate (++); intense (+++).

### Statistical analysis

The animals’ body weights, serum levels of albumin and tacrolimus and the results of rupture resistance of the three groups were initially analyzed by the Kolmogorov-Smirnov test. Since these variables had a normal distribution, the ANOVA test was used to compare the groups with the exception of serum tacrolimus levels, which were compared using the Student t test. The results of surgical wound infection and histopathology were analyzed using the exact chi-square test. The level of significance adopted in all tests was 5% (p<0.05). The analyzes were conducted using the SAS 9.3 program.

## RESULTS

In the immediate postoperative period and before receiving the medications, four animals in the control group and three in the experimental group died due to anesthetic complications or due to the surgical procedure. The other animals withstood the surgical intervention well, with no additional deaths during the study. Three animals in the GI had infection of the operative wound of the abdominal wall. No cases of infection were observed in GII or GC. As for the infection rate, there was no statistically significant difference between the three groups (p=0.175) and there was no dehiscence of the abdominal wall.

The distribution of the animals’ weights (Table 1) was statistically equivalent between the three groups (p=0.076).

The serum levels of albumin, in g/dl, of the animals in the three groups (Table 2) did not differ statistically (p=0.132).

There was no statistically significant difference in serum tacrolimus levels (Table 3) between animals in groups GI and GII (p=0.069).

Regarding the mean values of resistance to rupture of the abdominal wall scar, in Kgf, of the animals of the three groups (Table 4), no significant differences were observed between them (p=0.206).

**TABLE 1:** Distribution of body weights (g) of the three groups 10 days before the surgical procedure

Animal number	GROUPS		
	GC	GI	GII
1	240	206	210
2	219	182	278
3	205	196	198
4	252	244	315
5	219	366	354
6	260	399	364
7	230	340	320
8	208	409	401
9		341	364
10		330	376
11		361	
Mean value	229.13	306.72	318.00

**TABLE 2:** Serum albumin levels, in g/dl, of animals in the three groups on the day of sacrifice

Animal number	GROUPS		
	GC	GI	GII
1	2.8	2.3	2.8
2	2.8	3.0	2.9
3	2.9	2.8	3.1
4	2.9	2.8	2.4
5	3.0	2.4	3.0
6	2.9	2.8	2.7
7	2.8	2.4	2.9
8	3.0	2.6	2.9
9		2.9	2.9
10		2.8	2.9
11		2.9	
Mean value	2.89	2.70	2.85

**TABLE 3:** Tacrolimus levels in whole blood, in ng/ml, of animals in groups GI and GII

Animal number	GROUPS		
		GI	GII
1		0.6	0.2
2		0.2	0.2
3		0.1	0.1
4		0.2	0.4
5		0.6	0.1
6		0.2	0.2
7		0.4	0.5
8		0.5	0.1
9		0.5	0.4
10		0.6	0.2
11		0.3	
Mean value		0.38	0.24

**TABLE 4:** Values of resistance to rupture (Kgf) of the abdominal wall scar of the three groups

Animal number	GROUPS		
	GC	GI	GII
1	0.92	0.76	1.00
2	0.92	0.77	1.29
3	0.50	1.08	1.26
4	1.63	0.32	0.25
5	1.10	0.11	0.72
6	0.49	0.93	0.48
7	1.15	0.99	0.35
8	1.24	0.40	0.59
9		0.57	0.84
10		0.82	0.85
11		0.24	
Mean value	0.99	0.64	0.76

The results of the microscopic examination of the abdominal wall wound of the animals in the three groups are listed in Table 5. Most of the three groups showed a predominance of signs of inflammatory process, which varied from mild to intense, with the exception of one animal in the GI group. Four of the GI showed focal necrosis, three of moderate and one of mild intensity. Focal necrosis was observed in only one animal in the GII, being classified as mild in intensity. No animal in the GC presented focal necrosis. With the exception of one animal from GI and two from GII, all the others presented fibroblastic proliferation, which varied from mild to intense. All animals in the three groups showed vascular neoformation, which varied from mild to intense. The presence of fibrosis was observed in all animals in the three groups, with intensities varying from mild to intense.

When the comparative analysis of the findings of the inflammatory process of the abdominal wall of the three groups was carried out, there was no statistically significant difference (p=0.675). No statistical difference was observed between the three groups regarding focal necrosis (p=0.328), fibroblast proliferation (p=0.832), vascular neoformation (p=0.777) and fibrosis (p=0.053).

**TABLE 5:** Histopathological findings of animals in the three groups

Animal number	HISTOPATHOLOGICAL FINDINGS*														
	GC					GII					GII				
	A	B	C	D	E	A	B	C	D	E	A	B	C	D	E
1	+	-	++	++	++	+++	++	+++	+++	++	+	-	-	++	+
2	++	-	+	++	+	+	-	+	+	+	++	-	+++	++	++
3	+	-	+	++	++	++	-	+++	+++	++	+	-	-	+	+
4	+	-	++	++	++	+	-	+	+	+	+++	-	++	+++	++
5	+	-	+	+	++	+++	++	++	++	++	+	-	++	++	+
6	++	-	++	++	++	++	-	++	+++	++	++	+	+	++	++
7	++	-	+++	+++	++	-	-	-	+	+	+	-	+	+	+
8	++	-	++	++	++	++	-	++	++	++	++	-	++	+++	+++
9						++	+	+++	++	+++	++	-	+++	++	++
10						+++	++	+++	+++	+++	+	-	+	+	+
11						+	-	+	++	++					

*Classification of the findings in four degrees: absent (-); light (+); moderate (++); and intense (+++); A=inflammatory process; B=necrosis; C=fibroblast proliferation; D=vascular neoformation; E=fibrosis

## DISCUSSION

Despite advances in surgical techniques and perioperative care, the complications associated with healing of the abdominal wall remain a clinical and surgical challenge. After organ transplantation, the frequency of dehiscence and incisional hernia varied between 3.6-34%^6^ ^,^ ^18^ ^,^ ^19^, with an incidence rate similar to laparotomies for other indications^2^ ^,^ ^3^. Multiple predisposing factors were related to the development of early or late dehiscence of the abdominal wall wound^9^ ^,^ ^12^ ^,^ ^17^. In patients undergoing kidney or liver transplantation, the risk factors were similar to those undergoing abdominal surgical procedures not related to transplants^18^ ^,^ ^24^.

In the present study, the choice of the combination of immunosuppressive agent tacrolimus (calcineurin inhibitor) and mycophenolate mofetil (antiproliferative) was based on therapeutic advantages, being the most widely used combination in recent years for prophylaxis of transplant organ rejection^5^. The doses of tacrolimus and mycophenolate mofetil used for rats were similar to the usual clinical doses in patients undergoing organ transplants, ranging between 0.10-0.30 mg/kg of body weight per day and 1-3 g per day, respectively^1^ ^,^ ^11^. Only in GI, the dose of tacrolimus was higher than the usual clinical doses, but similar to that used in several experimental studies^20^ ^,^ ^26^.

Although there was no statistically significant difference, three animals from the GI, which received mycophenolate mofetil 20 mg/kg/day associated with tacrolimus 1 mg/kg/day, had wound infection. Several authors have shown that surgical wound infection was one of the most frequent complications in patients undergoing kidney transplantation^6^ ^,^ ^28^. In addition, surgical wound infection was the most frequent predisposing factor in the development of dehiscence and incisional hernia^8^. Animals treated with mycophenolate mofetil would be more prone to complications from the surgical wound due to their antiproliferative effect, as these agents act as inhibitors of fibroblast proliferation and cell signal transduction mediated by cytokine^1^ ^,^ ^23^.

In the present study, as the mean values ​​of resistance to rupture and the histopathological findings of the surgical scars of the abdominal wall did not differ significantly between the GC and the two treated groups (GI and GII), it was indicated that there was no interference from unfavorable factors in the healing process of the abdominal wall, including the absence of clinical repercussions, such as the non-development of an early complication (evisceration). Therefore, changes in the healing process, if any, can be repaired by the body, and are likely to be less severe.

Therefore, based on the results of the present study, treatments with the association of tacrolimus and mycophenolate mofetil did not significantly change the values ​​of resistance to rupture and the histopathological findings of the animals’ abdominal wall in this surgical model that sought to reproduce or simulate the clinical conditions observed in kidney transplantation.

In several clinical papers, it was not observed that regimens with tacrolimus^1,4,19^ and mycophenolate mofetil^23^ increased the risk of surgical wound complications when compared with other immunosuppression regimens in kidney transplantation. Valente et al.^23^ demonstrated that treatment based on the association of mycophenolate mofetil/tacrolimus/prednisone had a significantly lower rate of surgical wound complications (2.4%) than the group treated with sirolimus/tacrolimus/prednisone (43.2%). In a prospective, randomized clinical trial with comparative analysis of two immunosuppression regimes, tacrolimus/mycophenolate mofetil/prednisone vs. sirolimus/mycophenolate mofetil/prednisone, Dean et al.^1^ found that the rate of surgical wound complications was also significantly lower in the group based on tacrolimus (8%) compared to that of sirolimus (47%). In that study, complications from the surgical wound included infection, dehiscence and hernia of the abdominal wall. Clinical results obtained by different authors reinforce the experimental findings of the present study that the association of tacrolimus and mycophenolate mofetil does not interfere in the wound healing process.

The experimental studies available in the literature have analyzed the individual effects of tacrolimus and mycophenolate mofetil on the healing of different types of tissues and with contradictory results^13^ ^,^ ^15^ ^,^ ^20^ ^,^ ^25^ ^,^ ^26^. Willems et al.^26^ showed that the isolated use of tacrolimus did not produce adverse effects on the healing process during the first postoperative week. These authors evaluated the effects of tacrolimus in rats submitted to median laparotomy, followed by anastomoses in the terminal ileum and colon, which received tacrolimus subcutaneously at doses of 0.5, 2.0 and 5.0 mg/kg/day, postoperative until the date of sacrifice. The abdominal wall wound and both anastomoses were analyzed, but there was no statistically significant difference in the resistance to rupture of the abdominal wall and anastomoses between the groups that received tacrolimus and the control in the analyzed periods (3^rd^, 5^th^ and 7^th^ postoperative days). In contrast, Raptis et al.^15^ evidenced the promotion of the healing process of colic anastomosis based on significant increases in rupture intraluminal pressure and tissue hydroxyproline concentration in the group that received tacrolimus at a dose of 0.1 mg/kg/subcutaneously compared to the control group on the 4^th^ and 8^th^ postoperative days. Similarly, Kiyama et al.^10^ demonstrated that subclinical and therapeutic doses of tacrolimus improved the early stage of the healing process of colon anastomosis, as evidenced by the increased resistance of the anastomosis to rupture pressure compared to the control group on the 4^th^ day after operative. In the microscopic evaluation of the colic anastomosis, the authors also observed that the structure of the layers of the intestinal wall was better preserved, including the muscularis mucosa, the submucosa and the proper muscle, where the anastomosis was replaced by granulation tissue.

On the other hand, Schäffer et al.^21^ found that the values ​​of rupture intraluminal pressure and collagen content in colon anastomoses were statistically equivalent in groups of rats that were treated with subcutaneous injections of 2.0 or 5.0 mg/kg/day of tacrolimus and sacrificed on the 5^th^ postoperative day. However, in the same experiment, the authors observed statistically significant reductions in the hydroxyproline content, in the collagen deposit index and in the resistance to rupture of the skin wound in the animals that received 2.0 mg/kg/day of tacrolimus and that were sacrificed on the 10^th^ postoperative day compared to the control group. Based on the results, the authors concluded that high doses of tacrolimus would be needed to affect the healing process of the dermis and that different tissues demonstrated distinct susceptibility to immunosuppressant doses or that the restoration of the intestinal wound seems to evolve earlier compared to the injury of skin.

In order to evaluate the effects of the administration of 25 mg/kg/day of mycophenolate mofetil intraperitoneally on the healing of colic anastomosis in rats, Zeeh et al.^29^ showed that mycophenolate mofetil affected the surgical wound repair process, translated into a significant reduction in rupture pressure and proliferation rate compared to the control group on the 2^nd^ and 4^th^ postoperative days. Concordant results were obtained by Inglin et al.^7^, who demonstrated that the rupture pressures of the colon anastomosis were significantly lower in the group of animals treated with mycophenolate mofetil compared to the control group on coincident sacrifice dates. Sikas et al.^22^ reported that the values ​​of rupture pressure in colonic anastomoses were significantly lower in animals treated with 40 mg/kg/day of mycophenolate mofetil and sacrificed on the 3^rd^ and 7^th^ postoperative days, but with no statistically significant difference in the animals sacrificed on the 14^th^ day.

The present study evaluated the effects of the combination of tacrolimus and mycophenolate mofetil on the healing of the abdominal wall in an experimental model that simulates the conditions of a kidney transplant. Despite the limitations in relation to the number of animals and the high perioperative mortality, this model is similar to daily clinical practice, where these immunosuppressants are administered in combination and started early in the postoperative period.

## CONCLUSION

The immunosuppression schemes used associated with the renal ischemia-reperfusion phenomenon do not induce significant weakness in the surgical scar of the abdominal wall in rats.

## References

[1] Dean G, Lund WJ, Larson TS, Prieto M, Nyberg SL, Ishitani MB (2004). Wound-healing complications after kidney transplantation a prospective, randomized comparison of sirolimus and tacrolimus. Transplantation.

[2] Diener MK, Voss S, Jensen K, Büchler MW, Seiler CM (2010). Elective midline laparotomy closure the INLINE systematic review and meta-analysis. Ann Surg.

[3] Fikatas P, Schoening W, Lee JE, Chopra SS, Seehofer D, Guckelberger O (2013). Incidence, risks factors and management of incisional hernias in a high volume liver transplant center. Ann Transplant.

[4] Grim SA, Slover CM, Sankary H, Oberholzer J, Benedetti E, Clark NM (2006). Risk factors for wound healing complications in sirolimus-treated renal transplant recipients. Transplant Proc.

[5] Halloran F (2004). Immunosuppressive drugs for kidney transplantation. N Engl J Med.

[6] Humar A, Ramcharan T, Denny R, Gillingham KJ, Payne WD, Matas AJ (2001). Are wound complications after a kidney transplant more common with modern immunosuppression. Transplantation.

[7] Inglin RA, Baumann G, Wagner OJ, Candinas D, Egger B (2008). Insulin-like growth factor I improves aspects of mycophenolate mofetil-impaired anastomotic healing in an experimental model. Br J Surg.

[8] Irvin TT, Stoddard CJ, Greaney MG, Duthie HL (1977). Abdominal wound healing a prospective clinical study. Br Med J.

[9] Israelsson LA, Jonsson T (1996). Incisional hernia after midline laparotomy a prospective study. Eur J Surg.

[10] Kiyama T, Tajiri T, Tokunaga A, Yoshiyuki T, Barbul A (2002). Tacrolimus enhances colon anastomotic healing in rats. Wound Repair Regen.

[11] Knight SR, Russell NK, Barcena L, Morris PJ (2009). Mycophenolate mofetil decreases acute rejection and may improve graft survival in renal transplant recipients when compared with azathioprine a systematic review. Transplantation.

[12] Mehrabi A, Fonouni H, Wente M, Sadeghi M, Eisenbach C, Encke J (2006). Wound complications following kidney and liver transplantation. Clin Transplant.

[13] Paul GM, Tambara R, Repka JC (2014). Qualitative analysis of the deposit of collagen in bladder suture of rats treated with tacrolimus combined with mycophenolate-mofetil. Int Braz J Urol.

[14] Piazzese E, Montalti R, Beltempo P, Bertelli R, Puviani L, Pacilè V (2004). Incidence, predisposing factors, and results of surgical treatment of incisional hernia after orthotopic liver transplantation. Transplant Proc.

[15] Raptis D, Mantzoros I, Pramateftakis MG, Despoudi K, Zaraboukas T, Koliakos G (2012). The effects of tacrolimus on colonic anastomotic healing in rats. Int J Colorectal Dis.

[16] Raptis D, Pramateftakis MG, Kanellos I (2018). Our 20-year experience with experimental colonic anastomotic healing. J Med Life.

[17] Riou J, Cohen JR, Johnson H (1992). Factors influencing wound dehiscence. Am J Surg.

[18] Roine E, Bjork IT, Oyen O (2010). Targeting risk factors for impaired wound healing and wound complications after kidney transplantation. Transplant Proc.

[19] Santangelo M, Clemente M, Spiezia S, Grassia S, Di Capua F, La Tessa C (2009). Wound complications after kidney transplantation in nondiabetic patients. Transplant Proc.

[20] Schäffer MR, Fuchs N, Proksch B, Bongartz M, Beiter T, Becker HD (1998). Tacrolimus impairs wound healing a possible role of decreased nitric oxide synthesis. Transplantation.

[21] Schäffer M, Fuchs N, Völker J, Schulz T, Kapischke M, Viebahn R (2005). Differential effect of tacrolimus on dermal and intestinal wound healing. J Invest Surg.

[22] Sikas N, Imvrios G, Takoudas D, Gakis D, Papanikolaou V (2006). Mycophenolate mofetil impairs the integrity of colonic anastomosis. J Surg Res.

[23] Valente JF, Hricik D, Weigel K, Seaman D, Knauss T, Siegel CT (2003). Comparison of sirolimus vs mycophenolate mofetil on surgical complications and wound healing in adult kidney transplantation. Am J Transplant.

[24] Vardanian AJ, Farmer DJ, Ghobrial RM, Busuttil RW, Hiatt JR (2006). Incisional hernia after liver transplantation. J Am Coll Surg.

[25] Willems MC, Hendriks T, Lomme RM, de Man BM, van der Vliet JA (2016). The effect of mycophenolate mofetil on early wound healing in a rodent model. Transplant Direct.

[26] Willems MCM, Van der Vliet JA, Lomme RM, Hendriks T (2013). Tacrolimus does not affect early wound healing in a rodent model of bowel anastomosis and abdominal wall closure. Plos One.

[27] Winkler M, Ringe B, Baumann J, Loss M, Wonigeit K, Pichlmayr R (1994). Plasma vs whole blood for therapeutic drug monitoring of patients receiving FK 506 for immunosuppression. Clin Chem.

[28] Wszola M, Kwiatkowski A, Ostaszewska A, Górski L, Kuthan R, Sawicka-Grzelak A (2013). Surgical site infections after kidney transplantation - where do we stand now. Transplantation.

[29] Zeeh J, Inglin R, Baumann G, Dirsch O, Riley NE, Gerken G (2001). Mycophenolate mofetil impairs healing of left-sided colon anastomoses. Transplantation.

